# Same-Day Regimen as an Alternative to Split Preparation for Colonoscopy: A Systematic Review with Meta-Analysis

**DOI:** 10.1155/2019/7476023

**Published:** 2019-02-28

**Authors:** Cristina Bucci, Fabiana Zingone, Pietro Schettino, Clelia Marmo, Riccardo Marmo

**Affiliations:** ^1^Gastroenterology Unit, University of Salerno, Italy; ^2^Gastroenterology and Endoscopy Unit, L. Curto Hospital, Polla, Italy; ^3^Gastroenterology Unit, Department of Surgery, Oncology and Gastroenterology, University of Padua, Padua, Italy; ^4^Department of Clinical Medicine and Surgery, University of Naples Federico II, Naples, Italy; ^5^Division of Surgical Digestive System, University Hospital Second University of Naples, Italy

## Abstract

**Background:**

Split bowel preparation is the best regimen for colonoscopy. However, the same-day regimen can represent a valid alternative, but its use is limited by concerns about its cleansing ability, and to date, no convincing data support its use for routine colonoscopies.

**Aim:**

To evaluate the cleansing, compliance, and adverse event rates of the same-day compared to the split regimen.

**Results:**

A systematic literature search and meta-analysis was performed. Ten studies were included for a total of 1807 patients (880 in the same-day group and 927 in the split group). Overall, 85.3% patients in the same-day group vs. 86.3% in the split group had an adequate cleansing. Compliance was high for both, although patients were more compliant with the split than with the same-day prep (89.7% for same-day vs. 96.6% for split regimen). Sleep disturbance was more frequent in the split group, while nausea and vomit were more frequent in the same-day group. In the subgroup analysis, polyethylene glycol obtained a better cleansing rate when given as a split dose, with similar compliance and adverse events rates with both regimens.

**Conclusion:**

Split and same-day regimens are both useful in bowel cleaning before colonoscopy with a different pattern of adverse events and better compliance for split preparations. Endoscopists can consider the same-day preparation as a valid alternative, especially when the split preparation does not fit the patients' needs.

## 1. Introduction

Colonoscopy is considered the gold standard for the screening of colorectal cancer for its ability to identify and remove precancerous lesions [1]. Nonetheless, some adenomas or even cancers are missed during colonoscopy with a significant impact on the development of interval cancers [2, 3]. Reasons for this flaw detection rate may depend upon the operator's insufficient experience, inaccurate mucosal inspection, short withdrawal time, and substandard cleaning of the colon [4, 5]. Recent guidelines suggest using the split preparation for colonoscopy for its higher cleaning rate [6, 7] compared to the traditional previous-day regimen; however, concerns on the optimal bowel preparation still remain as some patients have an inadequate bowel cleansing also after this type of preparation.

Regardless of the regimen adopted, one of the critical factors for a clean colon is the time lapse between the end of the purge assumption and the beginning of colonoscopy. Ideally, this interval should not be longer than 5 hours to optimize the effects of the laxative [7, 8]. In this view, the same-day preparation has a shorter time lapse, providing the biological plausibility for an improved cleansing ability versus the split method, but it is recommended only for afternoon exams, as published studies were underpowered or provided controversial results.

Therefore, we aimed to evaluate the rate of adequate colon cleansing prior to colonoscopy by comparing the split regimen, the actual standard of care, to the same-day regimen. The secondary endpoints were to assess the rate of compliance and adverse events in the two groups.

## 2. Methods

### 2.1. Study Selection

We performed a systematic review of all the published articles (1960-2015) in which the same-day regimen was compared to the split one, following the methods as described in Bucci et al. [7]. Abstracts were included if the following inclusion criteria were fulfilled: (i) clinical trials, (ii) same-day vs. split regimens, or (iii) age > 18 years. Abstracts were excluded if not meeting the inclusion criteria or if the population was made up of a restricted group of patients, such as paediatrics or elderly patients. Of those selected, the full texts were obtained and the following data were extracted: study design, author, year of publication, patients' age, gender, diet prior to prep, time of colonoscopy, use of cathartics, compliance to the laxative (e.g., percentage of patients who took at least 75% of the prescribed dose), type, dose and regimen of prep, scale used to evaluate the colon cleaning, number of patients for each treatment arm with adequate/inadequate colon cleansing (grouping excellent-good vs. poor-fair), nausea, vomit, abdominal pain, bloating, and sleep disturbances. If one or more variables were not immediately inferable, principal investigators were contacted via e-mail. If the primary outcome was not available, the study was then excluded.

We assessed the studies quality using the Cochrane risk of bias assessment tool [9]. Studies were considered as having a low risk of bias if all risk domains were evaluated as low risk, a high risk of bias if at least one domain was assessed as high risk, or an unclear risk of bias if at least one domain was evaluated as vague without any high-risk domains.

### 2.2. Statistics

Percentage difference in the degree of colon cleaning between the same-day and the split preparation was the primary measure of the treatment effect. The meta-analysis was performed by computing the percentage difference using a random-effects model if heterogeneity was present. Quantitative analysis was performed on an intention-to-treat basis. Percentage difference and 95% confidence intervals (95% C.I.) for each treatment arm and pooled effect estimated were calculated. A forest graph was provided for the outcomes. Heterogeneity was calculated including the measures of consistency *I*^2^ for each pooled datum; a *p* value < 0.05 was considered as significant. Egger regression asymmetry test for publication bias and Funnel plot asymmetry were used for assessing the risk of bias at outcome level [10]. All measures were performed using the STATA software 11.2 version (StataCorp 2009, Stata Statistical Software: Release 11. College Station, TX: StataCorp LP).

## 3. Results

**Figure 1:**
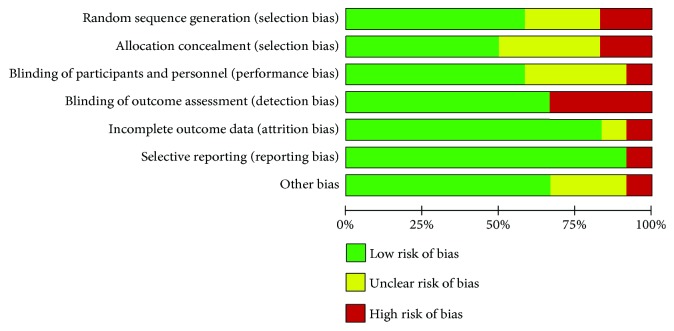
Risk of bias graph: a review of authors' judgments about each “risk of bias” item presented as percentages across all included studies.

**Figure 2:**
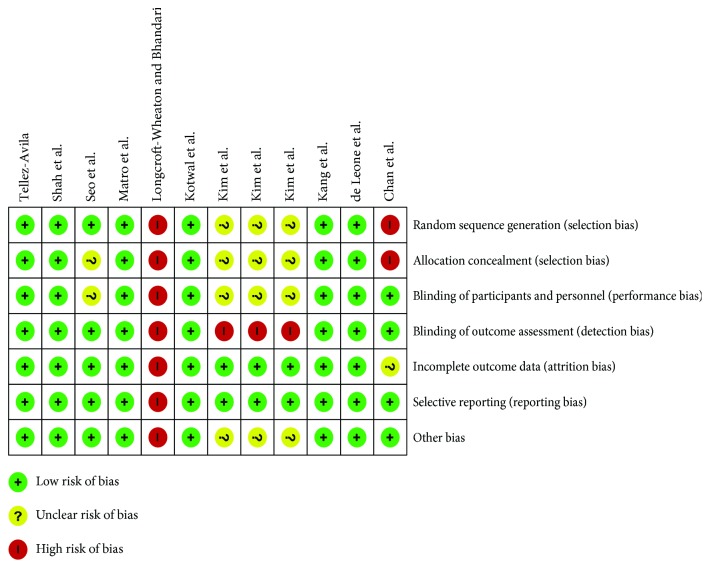
Risk of bias summary: a review of the authors' judgments about each risk of bias item for each included study.

Starting from 122 abstracts initially examined, ten full-text studies were retrieved, and 12 treatment arms were analysed for a total of 1807 patients [880 in the same-day regimen group (SDG) and 927 in the split regimen group (SG)]. The mean age of patients was 58.2 [±11.4 standard error (S.E.)] in the SDG and 57.8 (±11.9 S.E.) in the SG (*p* = 0.89), with a prevalence of 51.2% (95% C.I. 43.3-59.1) males in the SDG and 52.6% (95% C.I. 47.3-57.9) in the SG (*p* = 0.78). The studies' characteristics are summarized in Table 1. As for the bias, six trials were scored at low risk of bias [11–16], one trial was at unclear risk [17], and three trials were at high risk of bias [18–20] (Figure 1). For each study, bias was classified as represented in Figure 2.

A polyethylene glycol (PEG) laxative as same-day regimen was compared to split PEG into 8 studies [11–13, 15–17, 20], one study used same-day sodium picosulphate (PicoNa) versus split PEG [14], one compared PicoNa in both regimens [18], and one study tested same-day PEG vs. split 2 sachets of PicoNa or vs. split 3 sachets of PicoNa [19]. Six studies prescribed a 3-day low-residue diet before endoscopy [11, 14, 17–20] and in 2 studies a 1-day liquid diet was used [13, 15], while this information was unknown in the rest of the studies. Three studies used the Boston Bowel Preparation Scale to judge the degree of cleaning [11, 12, 20], 5 used the Ottawa [14–17, 19], and two used their own scale [13, 18]. Data on cleaning degrees were then categorized as adequate (good and excellent preparations) and inadequate (fair and poor preparations) cleaning.

**Figure 3:**
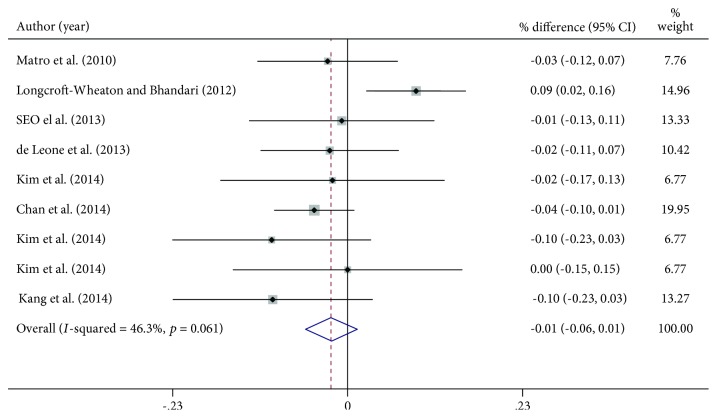
Percentage difference (expressed as rate difference, RD) of colon cleansing in patients assuming same-day or split bowel preparation. RD = rate difference.

Data on the number of patients with an adequate colon preparation and compliance were available in 9 out of 12 treatment arms. Overall, 569/667 patients in the same-day group vs. 619/717 in the split group had an adequate bowel cleansing with a pooled weighted rate difference (RD) of 2% [(95% C.I. -6% to 1%), *p* = 0.55; heterogeneity chi − squared = 13.83, *p* = 0.061; *I* − squared = 46.3%, Figure 3].

**Figure 4:**
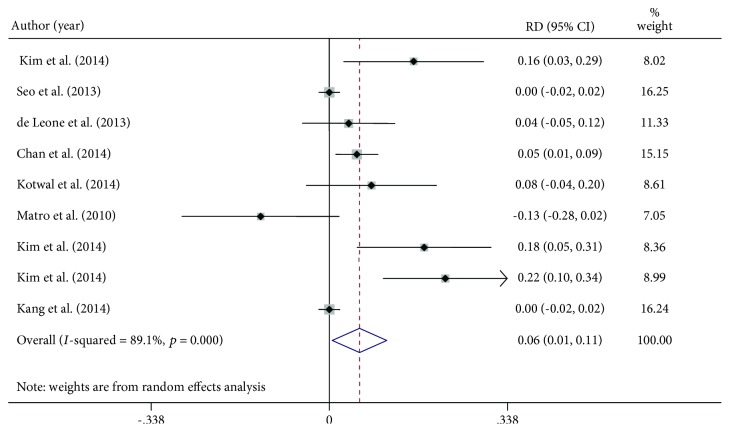
Percentage difference (expressed as rate difference, RD) of compliance of patients consuming same-day or split bowel preparation. RD = rate difference.

Data on compliance were available in 7 studies for a total of 586 patients in the same-day group and 674 in the split group. Although it was high for both regimens, patients were more compliant with the split preparation [RD 6% (95% C.I. 1-11%), *p* = 0.030; *I* − squared = 89.1%, *p* ≤ 0.001, Figure 4]. The wide heterogeneity can be attributable to a different magnitude of the observed effect among the studies [19, 20] but also to the effect direction [13]. In the study by Matro et al., all patients in the same-day preparation group underwent colonoscopy in afternoon sessions, making the preparation easier to drink in a prolonged time and thus more patient-friendly [13].

**Figure 5:**
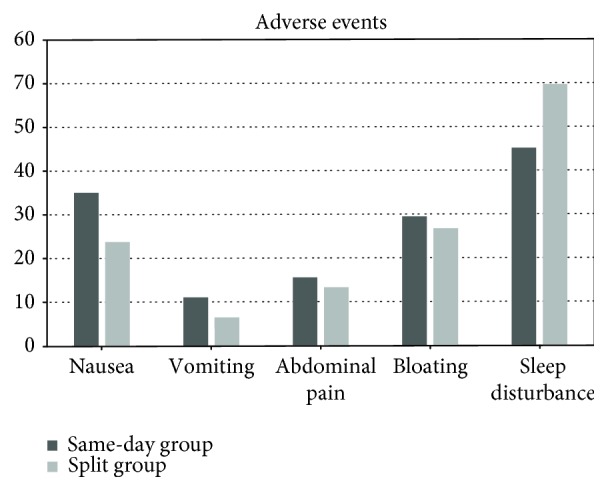
Distribution of adverse events in the same-day and split group, expressed as percentage.

All studies reported similar adverse events, which were extracted and compared (nausea, vomiting, bloating, abdominal pain/discomfort, and sleep disturbance). Nausea and vomit were more frequent in those who took the same-day prep [respectively, RD for nausea 10.5% (95% C.I. 2.4-18.6%), *p* = 0.011; RD for vomit 5% (95% C.I. 0.7-11%), *p* = 0.087]. In contrast, sleep disturbances were more frequent, even if not statistically significant, in the split regimen group [13.7% (95% C.I. -2.7 to 30%), *p* = 0.10]. No differences were noted for bloating and abdominal pain that were similar in the two groups [respectively, RD for bloating 2.3% (95% C.I. -5.9 to 10.6%), *p* = 0.624; RD for abdominal pain 1.7% (95% C.I. 4.6 to 8%), *p* = 0.595] (Figure 5).

### 3.1. Subgroup Analysis

#### 3.1.1. Same-Day PEG vs. Split PEG

Eight studies compared the PEG laxatives within different regimens. Three of them compared two low-volume preparations [13, 15, 20], two compared two high-volume solutions [16, 19], and three compared a low-volume same-day vs. a high-volume split preparation [11, 12, 17]. 652 patients were prescribed a same-day preparation and 633 a split one. The cleaning rate within the SDG was 90% (95% C.I. 87-93%) and 93% within the SG (95% C.I. 91-96%). With this laxative, a better, thus not statistically significant, cleaning rate was obtained with the split regimen [RD 3.3% (95% C.I. -7 to 0.5%), *p* = 0.086]. Compliance was comparable between the two regimens [RD -1.7% (95% C.I. -4.7 to 1.2%), *p* = 0.24]. Studies' heterogeneity was significant for compliance (24.42, *p* ≤ 0.001, *I* − squared = 71.3%), but not for the cleaning rate (52%, *p* = 0.972, *I* − squared = 0.0%). Adverse events were similar with both preparations except for nausea that was more frequent in the SDG [RD for nausea 7.7%, *p* = 0.020 (95% C.I. 1.2 to 14.1%); chi − squared = 15.30, *p* = 0.032; *I*-squared 54.2%].

#### 3.1.2. Same-Day Picosulphate vs. Split PEG

In the study by Kang et al., the authors tested a PicoNa morning only (97 patients) vs. 4 L PEG split preparation (99 patients) showing a slightly better, thus not statistically different, improvement in the cleaning rate with the PEG solution [respectively, 59/97 (61.5%) vs. 71/99 (71.3%), *p* = 0.13] [14]. Compliance was similar, but adverse events were overall lower with the same-day picosulphate (*p* ≤ 0.001), with a particular emphasis on sleep disturbances that were significantly fewer in the same-day group (*p* ≤ 0.001).

#### 3.1.3. Same-Day Picosulphate vs. Split Picosulphate

In this prospective nonrandomized study, Longcroft-Wheaton and Bhandari compared picosulphate in two different regimens [18]. Results showed that the same-day preparation was more effective than the split dose [130/132 (98%) same-day vs. 85/95 (89%) split, *p* = 0.007] with fewer adverse events (*p* ≤ 0.001), on equal compliance.

#### 3.1.4. Same-Day PEG vs. Split Picosulphate

In this three-arm randomized clinical trial, the cleaning efficacy of the same-day 4 L PEG (50 patients, group A) was compared to split doses of 2 picosulphate sachets (50 patients, group B) or to split doses of 3 picosulphate sachets (50 patients, group C) [19]. Patients with an excellent/good preparation were 82% (41/50) in the PEG group, 80% (40/50) in the two-sachet group, and 92% (46/50) in the three-sachet group (*p* = 0.325). Compliance was higher for picosulphate [RD -20.1% (95% C.I. -28.8 to -11.5%), *p* ≤ 0.001; heterogeneity chi − squared = 10.83, *p* = 0.650; *I* − squared = 0.0%]. In general, adverse events occurred less frequently with picosulphate preparations with the exception of sleep interruption that was uniformly distributed among groups [nausea RD = 37.1%, (95% C.I. 24.3 to 49.8%), *p* ≤ 0.001; vomit RD = 25%, (95% C.I. 16.1 to 33.9%), *p* ≤ 0.001; sleep disturbances RD = 16%, (95% C.I. 4.2 to -7.5%), *p* = 0.480; bloating RD = 46.5%, (95% C.I. 26.8 to 66.3%), *p* ≤ 0.001; and abdominal pain RD = 24.7%, (95% C.I. 13 to 36.5%), *p* ≤ 0.001].

## 4. Discussion

According to current guidelines, endoscopists should report the quality of the bowel preparation for screening colonoscopy and repeat within a year or less all those procedures classified as inadequate to visualize polyps < 5 mm in size [21, 22]. As a result, international societies published recommendations on bowel preparation for screening colonoscopies, agreeing on the superiority of the split preparation over other cleaning regimens [7, 23] and suggesting tailoring the type of purge to the patients' characteristics according to specific clinical scenarios (in patients, elderly, nephropathic, etc.). The split preparation is associated with higher compliance compared to the traditional previous-day regimen and a better quality of bowel preparation allowing for a substantial increase in the detection of advanced and serrated lesions [23, 24]. One of the main advantages of split preparation over the previous-day regimen is a shorter time lapse between the end of the preparation ingestion and the start of the procedure. It has been shown that the shorter the time lapse, the better the cleaning of the colon and hence the better mucosal visualization. On the other hand, the same-day preparation, which has by definition the shortest interval until the start of the procedure, has been relegated only for afternoon sessions of colonoscopy because the studies published so far are underpowered and provided conflicting results both for cleaning rate and compliance.

In our systematic review, the same-day preparation was compared to the split regimen with a total of 1807 patients included. Results showed that same-day regimens are not inferior to the split, as the overall cleaning efficacy was equivalent between the two and therefore should be taken into account as a valuable alternative regimen. Also, the subgroup analysis (i.e., per type of purge) confirmed the result of the primary analysis and suggested that PEG-based laxatives have their best use within the split regimen rather than the same-day. Conversely, from the patients' prospective, compliance was higher for split prep that was better accepted than the same-day. One could speculate that the reason for this different compliance could be sought in the type of laxative prescribed, as 8 out of 10 studies used a high- or low-volume PEG solution that is hard to drink in a few hours if compared with the same volume to be ingested in two separate doses, but definite conclusions cannot be drawn. Also, the compliance rate was heterogeneous among different studies as a result of a different magnitude and direction of the observed effect (while the cleaning rate was uniform in all but one study) [18]. Such heterogeneity may be due to the lack of use of clinical standards to describe the patients' experience. It would be advisable for future research to use dichotomous variables to describe the patients' experience as well as standardized scales for describing colon cleansing (such as Boston Bowel or Ottawa preparation scales instead of personal scales) to make results easily comparable.

When we compared the adverse events which occurred, sleep disturbance showed a nonsignificant trend toward significance in the split regimen group, while more cases of nausea and vomit were reported for the patients who took the same-day preparation. In our opinion, these differences could be attributable to the specific time-based characteristics of the regimen adopted rather than to the type of purge. Thus, they should be taken into account to optimize the patients' compliance when endoscopists prescribe the bowel preparation.

**Figure 6:**
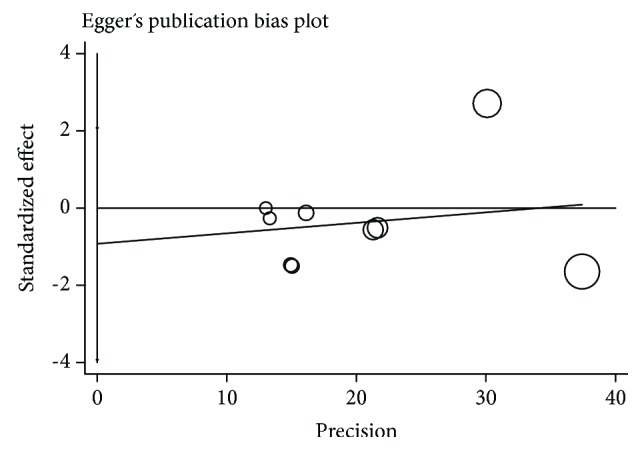
Egger's publication bias plot.

As for the publication bias, no significant asymmetry was observed in the meta-analysis, and neither significant was the Egger-Harbord regression (Figure 6).

The results of the meta-analysis show that the overall cleaning efficacy of the two regimens was comparable, but slightly better compliance favors the split prep.

As previously demonstrated, this datum confirms two critical elements: firstly, the cleaning efficacy is driven by the time (and thus a short time interval until the beginning of colonoscopy), more than by the type of laxative used. In other words, the second dose of laxative should be considered as the critical factor for an optimal bowel preparation, irrespective of the timing of the first dose, which consequently can be taken the evening before the exam or in the same morning, depending on the subject's need and endoscopy service organization; secondly, more efforts should be made to optimize the patients' compliance that should be regarded as a surrogate marker of quality in endoscopy. Patients' willingness is essential to get a good compliance and to increase participation in bowel screening programs as confirmed by the fact that two of the top ten patients' complaints for CRC screening are the bowel prep and the time spent for the test (e.g., missed work), more than the procedure itself [25, 26]. Also, in a recent publication by Radaelli et al. [27] who assessed patients' attitude toward two cleaning regimens (split vs. previous-day), the authors demonstrated that although split regimen was an independent predictor of adequate colon cleansing (OR 3.34) and polyp detection (OR 1.46) with no statistically different risk of travel interruption and fecal incontinence, appointment before 10:00 a.m. and travel time to endoscopy service > 1 h shift patients' preferences toward the previous-day regimen. Moreover, one should consider that patients might sometimes have personal or professional problems that make split preparation unsuitable in specific circumstances (e.g., evening dose unfeasible for work/personal situations). In our opinion, instead of using the same regimen for all patients, it could be advisable for endoscopy services to tailor the appropriate bowel preparation on the patients' needs and on the time of the scheduled colonoscopy, giving that overall the split and the same-day are comparable for the bowel cleaning. Still, patients should be clearly informed about pros (e.g., palatability and cleaning efficacy) and cons (adverse events, time needed, etc.) of the different regimens to improve their acceptance and reduce social barriers toward this screening tool.

In conclusion, although split preparation should be regarded as the best cleaning regimen for its high rate of efficacy and tolerability, the same-day preparation can be a valid alternative when the split preparation does not fit the patients' needs or when an afternoon colonoscopy is scheduled, provided that patients are informed on the different prevalence of side effects.

## Figures and Tables

**Table 1:** Characteristics of the studies included.

Author	Journal	Year	Laxative	Type of study	Scale	Same-day (total pts)	Split (total pts)	Compliance same-day	Compliance split	Same-day good prep	Same-day fair prep	Split good prep	Split fair prep
Matro et al.	AJG	2010	PEG vs. PEG	RCT	Own	61	54	52 (85.2%)	39 (72.2%)	56	5	51	3
Longcroft-Wheaton and Bhandari	J CLIN GASTR	2012	PicoNA vs. PicoNA	Prospective-NR	Own	132	95	UK	UK	130	2	85	10
de Leone et al.	WJGE	2013	PEG + bisacodyl vs. PEG	RCT	B	78	76	70 (89.7%)	71 (93.4%)	70	8	70	6
Seo et al.	Digestion	2013	PEG vs. PEG	RCT	O	97	100	97 (100%)	100 (100%)	72	25	75	25
Kim et al.	Scand J Gastr	2014	PEG vs. PEG	RCT	O	50	50	39 (78%)	47 (94%)	41	9	42	8
Kim et al.	Scand J Gastr	2014	PEG vs. PicoNA	RCT	O	50	50	39 (78%)	48 (96%)	41	9	46	4
Kim et al.	Scand J Gastr	2014	PEG vs. PicoNA	RCT	O	50	50	39 (78%)	50 (100%)	41	9	41	9
Kotwal et al.	J CLIN GASTR	2014	PEG vs. PEG	RCT	O	51	52	43 (84.3%)	48 (92.3%)				
Kang et al.	IR	2014	PicoNA vs. PEG	RCT	O	97	99	97 (100%)	99 (100%)	60	37	71	28
Chan et al.	WJG	2014	PEG vs. PEG	RCT	B	152	143	143 (94%)	142 (99.3%)	140	12	138	5
Tellez-Avila	DE	2014	PEG vs. PEG	RCT	B	59	61	UK	UK	UK	UK	UK	UK
Shah et al.	WJGE	2014	PEG vs. PEG	RCT	O	103	97	UK	UK	UK	UK	UK	UK

PicoNA = sodium picosulphate; PEG = polyethylene glycol; PTS = patients; RCT = randomized clinical trial; NR = nonrandomized; SCALE B = Boston Bowel Preparation Scale; O = Ottawa Bowel Preparation Scale; UK = unknown.
